# Coupling wastewater-based epidemiological surveillance and modelling of SARS-COV-2/COVID-19: Practical applications at the Public Health Agency of Canada

**DOI:** 10.14745/ccdr.v49i05a01

**Published:** 2023-05-01

**Authors:** Meong Jin Joung, Chand S Mangat, Edgard M Mejia, Audra Nagasawa, Anil Nichani, Carol Perez-Iratxeta, Shelley W Peterson, David Champredon

**Affiliations:** 1 National Microbiology Laboratory, Public Health Risk Sciences Division, Public Health Agency of Canada, Guelph, ON; 2 Dalla Lana School of Public Health, University of Toronto. Toronto, ON; 3 National Microbiology Laboratory, Wastewater Surveillance Unit, Public Health Agency of Canada, Winnipeg, MB; 4 Statistics Canada, Centre for Direct Health Measures, Ottawa, ON; 5 National Microbiology Laboratory, Public Health Agency of Canada, Guelph, ON

**Keywords:** COVID-19, SARS-CoV-2, wastewater, epidemiology, environmental surveillance, mathematical modelling, pandemic

## Abstract

Wastewater-based surveillance (WBS) of severe acute respiratory syndrome coronavirus 2 (SARS-CoV-2) offers a complementary tool for clinical surveillance to detect and monitor coronavirus disease 2019 (COVID-19). Since both symptomatic and asymptomatic individuals infected with SARS-CoV-2 can shed the virus through the fecal route, WBS has the potential to measure community prevalence of COVID-19 without restrictions from healthcare-seeking behaviours and clinical testing capacity. During the Omicron wave, the limited capacity of clinical testing to identify COVID-19 cases in many jurisdictions highlighted the utility of WBS to estimate disease prevalence and inform public health strategies; however, there is a plethora of in-sewage, environmental and laboratory factors that can influence WBS outcomes. The implementation of WBS, therefore, requires a comprehensive framework to outline a pipeline that accounts for these complex and nuanced factors. This article reviews the framework of the national WBS conducted at the Public Health Agency of Canada to present WBS methods used in Canada to track and monitor SARS-CoV-2. In particular, we focus on five Canadian cities—Vancouver, Edmonton, Toronto, Montréal and Halifax—whose wastewater signals are analyzed by a mathematical model to provide case forecasts and reproduction number estimates. The goal of this work is to share our insights on approaches to implement WBS. Importantly, the national WBS system has implications beyond COVID-19, as a similar framework can be applied to monitor other infectious disease pathogens or antimicrobial resistance in the community.

## Introduction

Epidemics caused by infectious pathogens are traditionally monitored through clinical surveillance of individuals. Wastewater-based surveillance (WBS) is an alternative epidemiological surveillance approach that consists of assessing the concentration of a pathogen of interest in wastewater to estimate its associated infection prevalence in a community. Wastewater-based surveillance has been integrated as part of poliovirus eradication initiatives since 2010 ((1)). In Canada, it has been used to monitor drug consumption and viral pathogens for seasonal viral load changes and inactivation by wastewater treatment processes ((2–6)). During the coronavirus disease 2019 (COVID-19) pandemic, WBS has attracted a lot of attention for surveillance of severe acute respiratory syndrome coronavirus 2 (SARS-CoV-2) (the virus that causes COVID-19) both in Canada and globally ((7)). Wastewater-based surveillance provides a complementary tool for clinical surveillance to detect and monitor trends of disease caused by SARS-CoV-2. In contrast to clinical surveillance of COVID-19 ((8)), WBS is not limited by underdiagnosis of asymptomatic individuals because most individuals infected with SARS-CoV-2 shed viral particles in their stools ((9,10)). Wastewater-based surveillance utilizes a pooled community sample from the catchment area of a sampling location to measure the levels of SARS-CoV-2 within the community ((11)). Multiple studies have shown that SARS-CoV-2 concentration measured in wastewater correlates with the real prevalence affecting the community living in the catchment area ((12–15)).

Wastewater-based surveillance garnered high interest during the emergence of the variant of concern Omicron in November 2021 ((16)). Its large number of genetic mutations compared to the previous circulating lineages conferred the variant a higher transmissibility and immune escape that fuelled a rapid growth of cases ((17)). Hence, during the Omicron wave, testing capacities in many countries, including in major Canadian cities, were overwhelmed, forcing the polymerase chain reaction (PCR)-testing of SARS-CoV-2 in clinical samples to be restricted to certain high-risk or vulnerable populations ((18)). Previous research demonstrated that SARS-CoV-2 was detected in 29%–100% of fecal samples in infected individuals ((19)) and that WBS detection of SARS-CoV-2 preceded confirmed clinical cases by 5–63 days ((11)), confirming WBS as 1) an alternative measure of disease prevalence, especially when clinical surveillance is limited by overwhelming demand or test-seeking behaviours and 2) an early indicator of COVID-19 presence to inform testing and public health strategies at the community level ((20)). Overall, WBS offers a non-invasive and low-cost method to estimate the community prevalence of COVID-19 that addresses the limitations of traditional clinical surveillance.

However, WBS is not free of biases and uncertainties. Wastewater-based surveillance can be influenced by various pre- and post-analytical factors, including methods of sample collection and storage, laboratory analysis protocol, engineering of the sewer network and wastewater treatment plants (WWTP), changes in weather conditions and data analysis procedures ((21–23)). Moreover, since WBS for SARS-CoV-2 is still evolving, there is a lack of standardized procedures to address these factors. Considering the potential sensitivity of WBS data to these factors, it is crucial to establish a pipeline that specifies standardized protocols and methodologies from sample collection to analysis to ensure the accuracy of WBS. While it may be impossible to control for some sources of uncertainty, minimizing their effects remains crucial. Importantly, there is a need to implement a framework to combine the results of WBS and clinical surveillance to clearly communicate the epidemiological findings to inform public health strategies ((24)).

In Canada, WBS is performed by laboratories at federal, provincial and municipal levels as well as by academic groups ((7)). The National Microbiology Laboratory (NML) at the Public Health Agency of Canada (PHAC) collates and analyses samples from multiple provinces to conduct WBS at the national level. The objective of this review article is to provide a comprehensive overview of the WBS pipeline at the NML and a framework to incorporate WBS and clinical surveillance to enhance the national surveillance of COVID-19. We describe how mathematical modelling can be utilized to facilitate the interpretation of WBS outputs. To increase the usefulness of the WBS data, we assess key factors that influence WBS signals at each step of the pipeline and methods to address them.

## Results

### Wastewater-based surveillance pipeline

**Figure 1:**
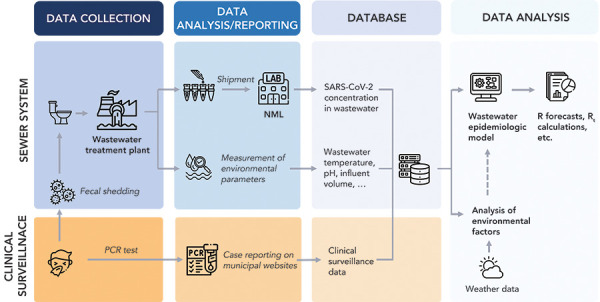
Wastewater-based surveillance data and analysis pipeline at Public Health Agency of Canada/National Microbiology Laboratory Abbreviations: NML, National Microbiology Laboratory; PCR, polymerase chain reaction; SARS-CoV-2, severe acute respiratory syndrome coronavirus 2

The Canadian national WBS program involves the collaboration of municipal WWTP and multiple government divisions and agencies, including Statistics Canada, NML and PHAC. The WBS pipeline was developed to streamline the WBS processes from sample collection to reporting in an accurate and timely manner (**Figure 1**).

### Data collection

**Figure 2:**
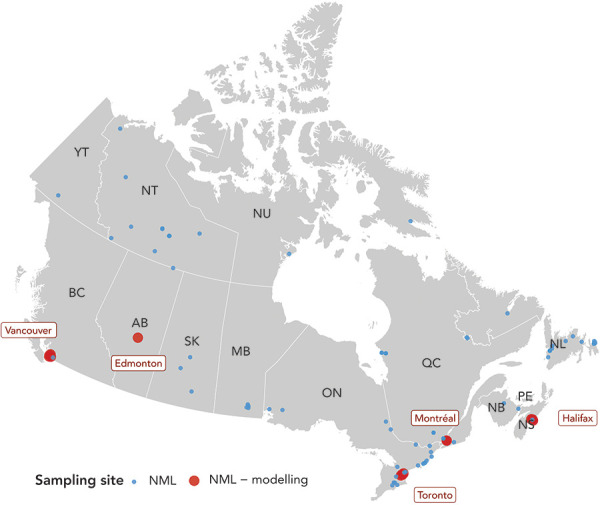
Wastewater-based surveillance of COVID-19 is actively conducted by federal, provincial and municipal governments and by academic institutions across Canada Abbreviations: AB, Alberta; BC, British Columbia; MB, Manitoba; NB, New Brunswick; NL, Newfoundland and Labrador; NML, National Microbiology Laboratory; NS, Nova Scotia; NT, Northwestern Territories; NU, Nunavut; ON, Ontario; PE, Prince Edward Island; QC, Québec; SK, Saskatchewan; WWTP, wastewater treatment plant; YT, Yukon

The Canadian Wastewater Survey, jointly led by Statistics Canada and PHAC, currently involves 102 WWTPs across Canada. We focus on 15 WWTPs of five cities—Vancouver, Edmonton, Toronto, Montréal and Halifax—where mathematical modelling is applied to analyze the trends of SARS-CoV-2 (**Figure 2**). The wastewater sampling in the five cities began in September 2020. The samples are collected approximately twice a week from the raw influents. Samples are collected before de-gritting in one WWTP in Edmonton, three WWTPs in Montréal and three WWTPs in Vancouver and post-grit removal in four WWTPs in Toronto and two WWTPs in Vancouver. Wastewater can be sampled using composite or “grab sample” methods. Grab sampling constitutes rapid sampling at a specific point in time, which represents the influent at that time; therefore, the results are more subject to changes in the influent flow of the day. Composite sampling involves collecting multiple samples using an automatic sampler during a set period (typically 24 hours) to represent the wastewater composition for that period. For the Canadian Wastewater Survey, the composite sampling method was used where automatic samplers collected wastewater samples during a 24-hour period. These samples were kept at 4°C and shipped to the NML in Winnipeg, Manitoba.

In addition to sample collection, wastewater quality and environmental parameters of the wastewater, such as influent daily volume, temperature and pH, are measured at the WWTP. The wastewater data from NML are collated together with the environmental parameters from each WWTP by Statistics Canada for data management and shared with PHAC/NML.

Clinical surveillance data are retrieved from the PHAC line list of COVID cases (an anonymized list of COVID cases, at the individual level, communicated by the provinces and territories to PHAC during the COVID-19 response) or publicly available sources on municipal websites for each city in cases where the PHAC line list does not have sufficient spatial resolution. When available (e.g. Toronto, Vancouver), we collect data at the sub-municipal level to map the spatial location of the clinical reported cases with the catchment area of each WWTP. Weather-related environmental data, including amount of precipitation and snow on ground for each city, are obtained from Environment Canada.

### Laboratory analysis of SARS-CoV-2 concentration

The SARS-CoV-2 concentration was measured with two methods. The laboratory protocols for the two methods were described in detail by Nourbakhsh *et al.* ((25)). Briefly, before February 12, 2021, SARS-CoV-2 ribonucleic acid (RNA) was extracted from the liquid supernatant portion of clarified wastewater samples. However, early studies found that the solid portion of clarified wastewater samples yield a higher viral concentration ((26–28)). Therefore, after February 12, 2021, RNA extraction was performed on the solid pellet after clarification. The change in protocol improved the efficiency of RNA quantification.

### Data quality and sources of uncertainty

The WBS data are influenced by several factors, including environmental conditions, laboratory protocols and engineering of the WWTPs. Below, we summarize how environmental and laboratory factors can impact WBS data. This is still an area of active research, and many knowledge gaps remain.

Environmental factors such as precipitation or snowmelt have been described as critical factors that could influence viral signals in the wastewater ((29)). However, the impact of environmental factors could vary depending on the type of the sewer system serviced by a WWTP. There are two major types of sewer systems—combined and sanitary. Combined systems collect storm water from surface runoff and wastewater together within the same pipes. While combined systems would only collect wastewater as influent water to the WWTP during dry weather, wet weather or high precipitation events (including snowmelt) would increase the influent flow rate and dilute viral concentration present in the wastewater ((29)). In contrast, sanitary systems mostly separate storm water and sewage, which means the influent volume do not change significantly based on the weather, avoiding the dilution of the viral signal.

Combined systems are present in older parts of the cities monitored by PHAC. To ensure the quality of the WBS data, we investigated the potential mediating effects of precipitation on the WBS SARS-CoV-2 signal. Our quantitative analyses of environmental factors (manuscript in preparation) revealed that while some fluctuations in influent volume were recorded with changes in precipitation, they do not appear to significantly impact the SARS-CoV-2 concentration in wastewater for the dates and sites analyzed. Snowmelt has also been suggested to influence the SARS-CoV-2 signal in wastewater ((21,30)). Although some studies showed the influent volume increased during snowmelt season ((30–32)), there is a paucity of evidence that snowmelt events have a significant impact on the viral signal.

### Laboratory factors

Viral concentration measurement from a wastewater sample is a multi-step process, where each step can introduce a potential source of error. The duration and conditions of transport of the sample from the sampling location to the laboratory may impact the final concentration measurement of SARS-CoV-2. By their nature, wastewater samples are very “active”; i.e., there is a high degree of biological activity that causes the nature of the sample to change rapidly. In addition, the equipment and containers of the sampling system may be contaminated. Therefore, storing, transporting, and handling wastewater samples are critical to maintain their integrity and are potential sources of errors. Moreover, the complex and variable nature of wastewater requires the proper use of control samples to account for variations in the composition of wastewater and evaluate overall efficiency of the process. Failure to properly run these controls are other potential sources of error. Molecular detection by real-time quantitative polymerase chain reaction (RT-qPCR) testing may also be prone to errors (e.g. standard curve not updated, new viral mutations affecting the identification by primers). Hence, rigorous protocols to ensure consistency and reliability of SARS-CoV-2 concentration measurements from wastewater samples should be in place at this stage of the WBS pipeline. Guidance regarding such protocols is presented in detail in the **Supplemental material**.

### Normalization

As mentioned above, many factors can affect the viral concentration in wastewater. Ideally, those factors would be identified, measured and controlled for before communicating a “final” viral concentration in wastewater. Wastewater is a complex matrix that contains biological, chemical and physical factors that may affect the RNA concentration and/or detection. Wastewater not only contains domestic sewage, but may also have industrial/agricultural discharge and storm water depending on weather conditions ((33)). From these influents, the composition of wastewater may change in pH, chlorine and dissolved oxygen content, which may reduce the viral concentration ((23)). Moreover, transportation of wastewater through the sewage network involves fluctuations in wastewater temperature, flow rate, sedimentation/resuspension and travel time. For these reasons, it is unlikely to have a consistently smooth viral signal in wastewater, especially when monitoring small communities. However, several normalization approaches have been employed by different groups to address these uncertainties. Normalization is not yet standardized in WBS; even the word “normalization” may not be appropriate because it attempts to correct for various factors. Viral signal in wastewater should be controlled for 1) human fecal mass to account for population (e.g. using biomarkers like pepper mild mottle virus [PMMoV], crAssphage and ammonia); 2) environmental events (e.g. WWTP influent flow) and 3) transport and dispersion dynamics in the sewer (e.g. using metrics of particle suspension in wastewater). There is likely no global solution for controlling for these (and other) factors, as each sewer has unique specificities. Normalization is still an area of investigation at PHAC/NML, where collection of several normalizing variables (e.g. concentration of PMMoV, pH, mass of total solids in suspension) has been performed since the start of the federal WBS program.

### Wastewater epidemiologic model

**Figure 3:**
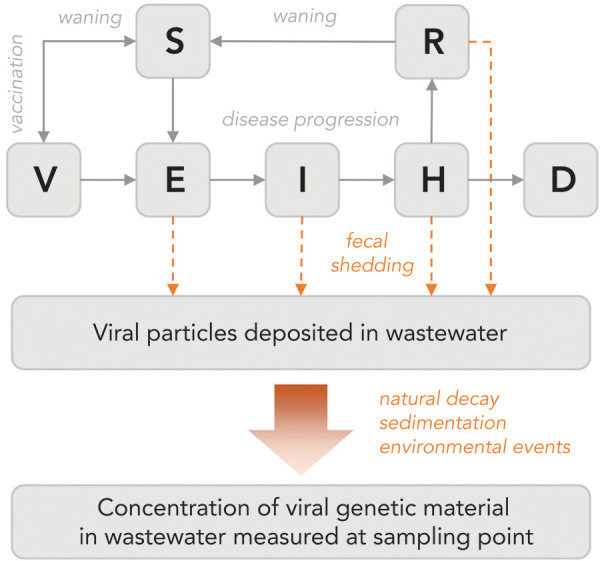
The wastewater epidemic model is based on standard mathematical modelling of disease spread in population^a^ Abbreviations: D, deceased; E, latent exposure; H, hospitalized; I, infectious; R, recovered; S, susceptible; V, immunization ^a^ Nourbakhsh *et al.* reference ((25))

A mathematical model that describes both SARS-CoV-2 transmission at the population level and SARS-CoV-2 concentration in the wastewater (by explicitly modelling fecal shedding) was developed at PHAC/NML ((25)) and implemented as a publicly available R package ((34)). A simple representation of this model, called the wastewater epidemic model (WEM), is shown in **Figure 3**.

Like other mathematical models, WEM provides a principled framework to estimate unobserved epidemiological parameters (e.g. prevalence, effective reproduction number R_t_) and to forecast cases, hospitalization and deaths. Importantly, WEM incorporates both the wastewater data and the traditional data based on clinical surveillance. These two data types, wastewater and clinical, can be used either in combination when more information is needed to triangulate the state of the pandemic, or as a substitute for one another when one of the two data source is missing or deemed inaccurate. We provide an example of the latter in the section analyzing the Omicron wave.

Because WEM integrates wastewater data, it translates the wastewater signal—that can be hard to interpret epidemiologically—into practical and well-known metrics for public health (e.g. prevalence, effective reproduction number). The lack of data and good understanding of the fate of viral RNA in the sewer prevented us from associating, *a priori,* the viral concentration measurement with the “true” prevalence level of infection in the catchment area of a WWTP. Thus, we were limited to estimating prevalence as if it was reported by clinical surveillance. This means we considered the historical data points of both the viral concentration in wastewater and the reported clinical prevalence to calculate their average ratio. We used this ratio to convert viral concentration into estimated “reportable” cases in WEM (i.e. reportable cases=ratio x viral concentration) ((25)). In other words, we did not try to estimate the reporting fraction. Although technically possible with WEM, we did not attempt to forecast hospitalizations or deaths because these data were not available to us at the sewer shed level (i.e. sub-municipal level), thus preventing us from fitting the model parameters associated with hospitalization and mortality. Hence, we limited our forecasts to reportable cases and the Supplemental material **Figure S1** shows our estimations for five Canadian cities.

We did not see significant differences in the forecasts produced by WEM, whether the concentration was normalized by PMMoV or not. Hence, we decided to simply use raw (unnormalized) SARS-CoV-2 concentration in wastewater since normalization is still an area of investigation at NML. The case forecasts are key indicators in planning public health actions because they predict the transmission of disease at the population level. We monitored the four-week forecast accuracy of WEM using log scores ((35)).

The effective reproduction number (R_t_) is another important measure that summarizes the current state of transmission dynamics. We show R_t_ estimates obtained from WEM in the Supplemental material **Figure S2**. These epidemiological indicators of virus transmission played an important role in the national COVID-19 surveillance, and modelling allows to incorporate information from WBS to enhance the estimation of these indicators.

### Wastewater-based surveillance reporting

Wastewater-based surveillance is, by nature, conducted locally—typically at the level of a municipality (sampling at a WWTP), a neighbourhood (sampling in a manhole) or an institution (e.g. hospital, university campus). When data from several sampling sites are available, it may be more relevant to aggregate the data to provide a trend indicator for a broader geographical area. A possible approach to aggregate viral concentrations in wastewater from different sites is to perform a weighted average where the weights represent the population sizes of each catchment area. Of course, the viral concentrations must be standardized beforehand.

To inform its analyses, PHAC aggregates WBS from samples collected at the wastewater treatment plants to municipal and national levels. PHAC analyzes WBS through the lens of modelling; hence, the weighted average aggregation is performed on the epidemiological metrics (e.g. forecasted incidence, R_t_) after fitting WEM to the data of each sampling site. In other words, we do not fit WEM to an aggregated wastewater signal. Wastewater-based surveillance is reported in combination with clinical surveillance and modelling forecasts to show the wastewater concentration and cases to date, and predictions based on WEM.

### Application to the analysis of the Omicron wave in Canada

The Omicron variant of SARS-CoV-2 was classified as a variant of concern on November 26, 2021 ((16)). By January 2022, over 90% of SARS-CoV-2 samples collected in Canada were identified as Omicron ((36)). Omicron spread rapidly across Canada, which prompted a change in testing policies to restrict PCR testing to high-risk or vulnerable populations in many jurisdictions to meet the overwhelming demand. This change likely led to an underestimation of disease burden by clinical surveillance. Importantly, case forecasts from models using the case data could no longer serve as reliable indicators to inform public health policies. In fact, in all five cities analyzed with WEM, wastewater viral loads increased concordantly with clinical cases, but the trends diverged with the implementation of PCR testing restrictions (Supplemental material, Figure S1). While clinical cases appeared to have peaked around the date of the restriction, wastewater signals remained elevated or continued to increase. The discordance between clinical surveillance and WBS during the Omicron wave emphasized the utility of WBS when clinical testing was restricted ((3)).

**Figure 4:**
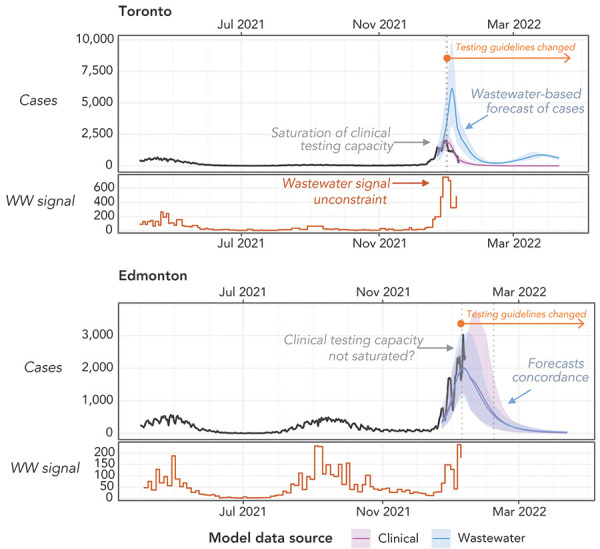
Example of model output interpretation during the Omicron wave Abbreviation: WW, wastewater

To assess the impact of the data source on case estimates and forecasting with WEM in absence of reliable clinical testing, the model was calibrated alternatively to clinical and WBS data. In addition, the model parameters representing the asymptomatic proportion and vaccine efficacy—assumed constant for all the waves before Omicron—were calibrated on available Omicron-specific data once they became available (e.g. early studies on vaccine effectiveness). After these adjustments were made, data from WBS, clinical surveillance and model forecasts were reported with epidemiological interpretations for internal monitoring of the national SARS-CoV-2 trends (Figure 1). The WEM provided estimates of reportable cases (i.e. clinical cases that would have been reported without PCR testing restrictions) using wastewater data only, in comparison with actual reported clinical cases, to assess the extent of under-reporting and the likelihood of having passed peak incidence of the wave. In **Figure 4**, we illustrate how modelling outputs were used in two different cities during the Omicron wave. In this example, WEM was fitted alternatively to clinical or wastewater data in Toronto (the largest city in Canada) and Edmonton (a medium-size city). The model suggests that under-reporting of cases in the former was more pronounced than in the latter. This modelling analysis of the Omicron wave, together with the estimates for five cities over a longer period presented in the Supplemental material, Figure S1. highlighted the limitations of clinical surveillance, especially after the change in PCR testing guidelines. From WBS, the under-reporting of cases was evident through the comparison of cases estimated from clinical surveillance and WBS. Moreover, WBS complemented the information from clinical surveillance including the timing of the peak and increasing/decreasing trends. Overall, the Omicron wave in Canada has allowed for an appreciation for the utility of WBS as an alternative approach to monitor SARS-CoV-2 transmission when clinical surveillance became overwhelmed and struggled to provide high quality data on disease prevalence trends.

## Discussion

### Limitations of wastewater-based surveillance in Canada

Currently, the wastewater-based modelling focuses on five major cities in Canada. While the combined catchment area of WBS for these five cities is about 23% of the Canadian population ((37)), it cannot provide a comprehensive overview of the SARS-CoV-2 trends with the limited scope of surveillance. It is not clear what minimum proportion of the population should be monitored through WBS to provide a reliable estimate of national prevalence. Monitoring large cities may be a good starting point to assess the intensity of transmission nationwide because most of the transmission likely occurs there. Although the expansion of the Canadian Wastewater Survey may increase the coverage of WBS, several challenges are anticipated given the geography and population distributions in Canada.

First, WBS in small or remote communities will require different sampling methods, such as sample collection from a septic tank, manhole, or lagoon, due to the absence of WWTPs in such areas. Although previous research has demonstrated that sampling from manholes, did not result in significant RNA decay ((38)), it poses as a logistical challenge. Moreover, our current modelling framework (WEM) is not adapted to analyze small populations, mainly because WEM is not a stochastic model.

Although still an area of active research, controlling for uncertainty in the viral signal in wastewater, such as fecal shedding dynamics and in-sewer RNA decay, is critical. Since the viral signal is meant to be used to inform public health, normalization may improve its accuracy in estimating the prevalence of infections. The uncertainty of the efficacy of normalization techniques, at PHAC/NML but also for many other groups, is currently a limitation that hampers the interpretation of WBS and an area of active research.

### Beyond COVID-19

The implementation of WBS as a routine surveillance tool has broader implications beyond COVID-19. Wastewater-based surveillance can also be used for monitoring respiratory pathogens other than SARS-CoV-2 (including influenza viruses, respiratory syncytial virus), sexually transmitted infections, antibiotic resistance and antibiotic use in the community ((39,40)). Importantly, the active research of WBS during the COVID-19 pandemic allowed for a better understanding of in-sewer factors, environmental factors and population dynamics that affect WBS and the development of mathematical modelling to estimate population prevalence of the health risk and its future predictions. However, we note that for any pathogen surveyed in wastewater, it is critical to understand its fecal shedding dynamics and in-sewer decay to improve estimates of infection prevalence in the community from viral concentration measured in wastewater. Unfortunately, there is a dearth of such clinical studies, even for SARS-CoV-2. While the expansion of WBS to other pathologies will require the development of novel laboratory assays, the current framework and knowledge of WBS and modelling with WEM will provide a strong foundation to facilitate the surveillance of other infectious pathogens.

### Next steps

While the present framework provides a comprehensive WBS pipeline for the current scope of national WBS, changes and improvements can be implemented to respond to the dynamic nature of the COVID-19 pandemic. A crucial step in further developing WBS is to standardize the surveillance data, including its measurement metrics and storage, across many laboratories. The Public Health Environmental Surveillance Open Data Model ((41)) is an initiative to develop an open data structure, including metadata and vocabulary, to support environmental surveillance such as WBS. PHAC is in the process of incorporating its national WBS into the Public Health Environmental Surveillance Open Data Model to augment its capacity to monitor multiple pathogens and geographical locations for WBS—facilitating the scalability of data analysis, thanks to its standardized data structure. In addition to incorporating data from concurrent WBS programs, WBS has the potential to expand to more geographical locations with diverse environments, such as remote or small communities. However, remote communities pose unique challenges because they often lack a WWTP and require alternative sampling methods for WBS. Hence, the framework may also expand to incorporate data analysis processes from these varying sources of WBS samples to standardize the analyses. Lastly, WBS can serve as an indicator of emerging variants of concern through SARS-CoV-2’s genome sequencing. Although this is currently conducted at NML, the epidemiological interpretations of the results are not yet incorporated in the WBS pipeline described here.

## Conclusion

Although WBS has previously been used to inform public health responses for other health risks, the COVID-19 pandemic stimulated an expansion of WBS to an unprecedented scale. As demonstrated during the Omicron wave, COVID-19 WBS has the potential to have high policy implications, especially when traditional epidemiological surveillance methods are curtailed. The present framework outlines the first national WBS of COVID-19 in Canada. In particular, the use of mathematical modelling is a critical tool to interpret WBS because it translates wastewater concentrations into prevalence for easier interpretation in public health settings. While WBS of COVID-19 provides unique information on the community spread of SARS-CoV-2, there remain many uncertainties and inconsistencies to be addressed in WBS data. The establishment of this framework will support further expansion and development of the WBS program, including monitoring other geographical areas and other pathogens.

## Supplemental material

These documents can be accessed on the Supplemental material file.
